# Cocaine

**Published:** 1891-12

**Authors:** F. A. Stevenson

**Affiliations:** Montreal.


ARTICLE VI.

### COCAINE.

BY F. A. STEVENSON, D. M. D., L. D. S., MONTREAL.

Cocaine is the alkaloid obtained from the *leaves* of a South American shrub called Erythroxylon coca, which is found chiefly on the eastern slopes of the Andes in Peru and Chili. The coca leaf resembles the tea leaf in shape, and has an astringent and bitter taste, and is used by the natives both as a medicine and a stimulant.

The alkaloid, when pure, occurs in the form of transparent prisms, of a bitter taste, and is only slightly soluble in water, but dissolves readily in ether, and fairly well in alcohol. There are several salts of cocaine used in medicine—the hydrochlorate (or muriate), the citrate and hydrobromate; there is also a wine of coca which has a very stimulating effect on the nerves. The hydrochlorate is the salt most generally used for producing local anæsthesia. It began to be used by dentists about 1884, principally to deaden the pain of extraction of the teeth. The usual course was to apply a 4 per cent. solution to the gum (many, also, injected it between the gum and the tooth), then, after waiting for five minutes to allow the cocaine to take effect, to extract the tooth. Very soon cases with very disagreeable constitutional symptoms were reported, such as severe cramps in the extremities, or fainting. I know of a case in which several teeth were extracted under the influence of cocaine, and partial paralysis of the tongue occurred which lasted for three days, and then gradually wore off. Of course, a natural revulsion against a drug which acted so capriciously followed, and its general use in the extraction of teeth has been given up. A few dentists, however, still continue to use cocaine. The late Dr. Whitten, of Boston, used it extensively, and claimed never to have had any unpleasant after-effects. He used a very strong solution of the hydrochlorate (20 per cent.), and injected 3 m into the socket of the tooth to be extracted, making three injections into as many sides (1 m into each. He then extracted at once, without allowing any time to elapse between the last injection and the extraction. Whitten's theory was, that the very small quantity of cocaine injected, and extracting immediately, prevented the alkaloid from being absorbed in a quantity sufficient to produce constitutional effects. I have found the anæsthetic effect from this method only partially successful, perhaps because I have only used it in cases where the patient was in a state of panic and afraid to take $H_2O$.

As applied to sensitive dentine, cocaine is practically useless, as it does not seem able to penetrate the dentine to any depth. According to one authority, it requires twenty minutes to take effect, which is more time than most of us would care to spend.

In the benumbing of partially devitalized pulps, cocaine is really very useful. It may be used freely in the strongest solutions, if care is taken to prevent any from being swallowed or getting into the mouth.

For some mysterious reason, drugs applied to the pulp of a fully formed permanent tooth are not apparently absorbed in the system.

By the use of cocaine, the pulp may be removed before it has become decomposed, and has still enough consistence to enable the nerve brooch to withdraw the pulp as a whole instead of piecemeal. The pulp cavity may be easily washed out and filled, without fear of any soreness afterwards.

I have found the following successful in every case, except in the treatment of two molars:—First apply the rubber dam to prevent any of the cocaine from being swallowed. Then uncover the pulp as much as possible, which does not cause very much pain in the majority of cases, the pulp being already somewhat exposed from decay. If the surface of the pulp is extremely sensitive, apply some of the crystals of the cocaine to it, and leave them there while getting your hydrochlorate ready. The needle of the syringe should have had the point ground down, and the temper should be drawn, so that it may be bent in any direction. The solution of cocaine used should be at least 20 per cent. strong. It is best to keep a supply of the hydrochlorate on hand, and make the solution as it is required, for it decomposes in a few days. Inject two minims. This will cause an unpleasant sensation for a moment, not amounting to pain. If there is still more feeling in the pulp, on applying the nerve brooch, repeat the injection, when the sensation will be destroyed, and the pulp may be removed. Immediately after the removal of the nerve there will be a considerable hæmorrhage, which, however, soon stops; the pulp cavity may then be washed out and filled at once. The whole operation may be done, and the tooth filled, within an hour, and it is the most satisfactory method of immediate root-filling that I have had anything to do with.

The two molars mentioned above as being unsuccessful were troublesome on account of the difficulty of getting at them with the hypodermic syringe, and also the narrowness of the buccal roots seemed to prevent the cocaine from taking effect on the pulp in them. It is important, in order to protect the needle of the hypodermic syringe, to wash it in clear $H_2O$ immediately after the operation, as the solution of cocaine seems to corrode the steel.

If anything that I have said should prove of use to any of the gentlemen here, the object of this little paper will have been attained.—*Dominion Dental Journal.*

